# Repatriation of South Sudanese refugees from the West Nile districts, Uganda. What do we learn from the process?

**DOI:** 10.4314/ahs.v22i2.17S

**Published:** 2022-08

**Authors:** Henry Komakech, Christopher Garimoi Orach

**Affiliations:** Makerere University, School of Public Health, Department of Community Health and Behavioural Sciences

**Keywords:** Refugee, repatriation, process of repatriation, South Sudan, west Nile, Uganda

## Abstract

**Introduction:**

Repatriation is a fundamental and often preferred solution to the refugee crisis around the world. This study explored the process of repatriation of the South Sudanese refugees from the West Nile districts in Uganda.

**Methods:**

This was a retrospective analysis of the process of repatriation of refugees in three west Nile refugee districts of Adjumani, Arua, and Moyo, Uganda. Both qualitative and qualitative data were collected.

**Results:**

The findings showed that several stakeholders were involved in the repatriation exercise including the government at central and district levels, United Nations agencies and non-governmental organizations, and refugee communities. The key steps undertaken during repatriation include information and education campaigns to promote returns focussing on security and socio-economic conditions in South Sudan and the facilitation of confidence-building visits in the areas of origin. During the repatriation exercise, key interventions provided included health care screening and treatment, and the provision of reintegration support services including the provision of food security items and cash allowances.

**Conclusions:**

The findings highlight the fundamental steps followed during a well-planned, structured, and effective repatriation of South Sudanese refugees from Uganda. Understanding repatriation requires an appreciation of how it is implemented to support offering an effective, durable, and lasting solution to refugees to promote their health and welfare.

## Introduction

Globally, an estimated 89 million people were forcibly displaced at the end of 2021. This included 21 million refugees and 48 million Internally Displaced Persons (IDPs)^1^. Low-income countries hosted the majority, 86% of the world's refugees^2^. The leading refugee-hosting countries around include Uganda, Turkey, Jordan, Pakistan, and Lebanon with over 20 million refugees. Africa hosts approximately 30% of the worlds displaced population. East Africa, the horn of Africa and the Great Lakes region hosted about 4.5 million refugees^3^. Several countries in the region host large numbers of refugees including Uganda 1,269,758, Democratic Republic of Congo 533,656, Ethiopia 841,285, Tanzania 306,025, Sudan 538,797, and Kenya 433,457, respectively^1^.

Uganda has been hosting refugees and asylum seekers since achieving its independence in 1962. Most refugees in the country are caught up in a protracted situation due to prolonged conflict in various countries. Most of the refugees in Uganda originate from neighbouring countries including the Democratic Republic of Congo, Rwanda, and South Sudan. Refugee influx into Uganda has been protracted and recurring over several years. During the 1960s, an estimated 86,000 Sudanese fled into Uganda^4^. Between 1993 - 1994, a second major influx of an estimated 100,000 south Sudan refugees fled into the west Nile region (Payne)^5^. By 1995, an estimated 170,000 - 210,000 Sudanese refugees were settled in Uganda^4^. By the end of 2020, there were an estimated 1.4 million refugees in the country.

Finding lasting durable solutions is an essential aspect of refugees protection and assistance^6^,^7^. Globally, the United Nations High Commission for Refugees (UNHCR) is mandated to provide assistance and protection to refugees^8^. Three primary durable solutions are promoted including voluntary repatriation, resettlement into a third country and local integration. Repatriation, however, remains the preferred solution for the UNHCR, and governments based on several international statutes. The statute of non-refoulement, codified in Article 33 of the 1951 UN Refugee Convention, forbids a state receiving refugees from forcibly returning them to a country where the cause for flight originated^9^. The principle is reinforced by Article 5 of the Organization of African Unity (OAU) 1969 Refugee Convention and the UNHCR's Executive Committee, conclusion of 1985 that affirms the need for voluntary repatriation^10^. Implementation of the principle is influenced by several factors. First, the host country's refugee laws and bureaucracies that determine its compliance with the international standards. Secondly, the international refugee regime and its influence on host governments' ability to accept refugees by provision of technical and financial assistance. Finally, developing countries hosting refugees may have low absorptive capacity in terms of resources and social receptiveness which will influence the decision to admit refuges^11^.

Several studies provide insights into the process of refugee repatriation. A review by^12^ examined the dynamics of flight, settlement in exile and resettlement. The findings show that while improvements in the country of origin should lead to repatriations, refugees are resistant to relocation and often avoid returns. Moreover,^13^ observed that whereas there are several approaches of achieving repatriation, refugees are often involved by organising their own returns. While,^14^ described how repatriation was promoted through the signing of tripartite agreements, informing refugees that it was safe to return, and provision of assistance. In a study of indigenous and non-indigenous Nicaraguan from Honduras and Costa Rica,^15^ reported that the majority of refugees returned from exile due mainly to the host governments immigration and employment policies and favourable stakeholders attitudes. Whereas a study by^16^ noted that refugees often evaluate their identity both as insiders and foreigners and the uncertainty of their status as essential determinants for the scale, process and success of returns. However,^17^ observed that the “kinetics” of the original flight, forms of social diversity, level of politicization, and the personality of a refugee are crucial in the decision of how and when to be repatriated. Despite these studies, there remains a dearth of evidence to illuminate the process of refugee repatriation in developing countries. This paper examined the process of refugee repatriation by analysing the specific steps taken to inform future repatriation policies, practice and procedures.

## Context of repatriation of south Sudanese refugees

The repatriation of the south Sudanese refugees was facilitated by two key interrelated events. First, the signing of the Comprehensive Peace Agreement (CPA) between the Government of the Republic of Sudan, and Sudan`s Peoples Liberation Movement/Army (SPLM/A). The signing of the CPA ended decades of conflict between the government of the republic of Sudan and SPLM/A. Significantly, the CPA led to improvement in security condition in many areas of South Sudan. This enabled the key party's' to consider and commence the process of repatriation of the refugees.

Secondly, the signing of the tripartite agreement between the UNHCR, the governments of Uganda, and Sudan provided the legal and institutional frameworks for the repatriation exercise. The tripartite agreement specified the roles of the different stakeholders and how the signatories had to commit to assisting the refugees to return in peace and dignity without any fear of persecution. The government of the Republic of Sudan had to provide guarantees to all refugees to return safely. The government of Uganda committed to providing settlement and security for all refugees including both those willing and unwilling to return. Furthermore, the agreement stipulated that all refugees had the right to return and start a normal and stable life in their communities. The agreement established structures and operational procedures for the UNHCR, governments and all other stakeholders to follow in organizing and supporting the voluntary repatriation of the refugees.

## Methods

### Study design and setting

This was a retrospective analysis of refugee repatriation conducted in the west Nile districts of Arua, Adjumani, and Moyo. The study analysed the numbers of refugees repatriated, places of origin, and how the process of repatriation was conducted. The west Nile region has been host to refugees from then Sudan now south Sudan since the 1990s. Refugees have been living in settlements in the districts of Moyo, Arua, and Adjumani interspersed amongst the host populations where they share social services (health and educational) and socio-economic resources. Currently, there are an estimated 1.4 south Sudanese refugees living in various settlements across the west Nile districts in Uganda^18^.

### Study population

The study population comprised civil administrators and managers, health service providers and project staff from Non-Governmental Organizations (NGOs) working with refugees particularly during the repatriation exercise. Respondents were purposefully selected in all the three study districts. These included key project staff working with UNHCR, NGO local and national staff of Office of the Prime Minister, refugee desk officers, repatriation nurses, district health officers, and local government officers in the districts of Arua, Adjumani Moyo. In addition, we reviewed records and reports of the repatriation of refugees from the UNHCR, Office of the Prime Minister, and the district local governments.

### Data collection procedure

Data were collected during the period June 2015 - December 2016. Qualitative data were collected using in-depth and key informant interviews and informal discussions. A total of 21 interviews were conducted with various respondents. Qualitative data were collected using a semi-structured interview guide. The interview guide included broad, open ended topic questions to allow in-depth discussions from different perspectives. Interview data were audio-recorded using a digital recorder after a respondent had consented to participate in the study. Interviews were conducted at respondent's places of work, in quiet and private locations. Respondents were encouraged to discuss the topics at length, and interviews were guided by probes. Data were collected from key informants with knowledgeable and had been involved in the process of repatriation. All interviews were conducted in English by the PI and research assistants.

Quantitative data were collected by reviewing the records and documents relating to the settlements of residence in west Nile, numbers of refugees repatriated, places of origin and how the process of repatriation was conducted. The documents reviewed were obtained from various organisations including the UNHCR, Office of the Prime Minister, African Humanitarian Action (AHA), and the district local government authorities of Arua, Adjumani, and Moyo respectively. Additional sources of data included reports, strategic and operational plans, policy documents, minutes of planning meetings and field notes related to the repatriation of refugees.

### Data management and analysis

Qualitative data management and analysis were guided by the grounded theory approach^19^. The authors read through the field notes to identify the steps in the processes of refugee repatriation. Interview transcripts and field notes were examined and categorized into themes. Transcripts were analysed to identify the issues brought out by the various key informants. Qualitative data were analysed manually. Quantitative data were analysed using Microsoft Excel computer programme.

## Results

### Steps undertaken in preparation for repatriation

The first step taken to prepare for repatriation was that the UNHCR and the GoU through the Office of the Prime Minister (OPM) organised mass sensitization campaigns to inform and enable refugees make free and informed decision about repatriation. Information campaigns were conducted through several approaches including meetings, mass media, and use of influential community members to promote the repatriation exercise. Sensitization took place in all the settlements and in various places including within the settlements, at the registration and verification centres in all three districts. The sensitization campaigns were conducted in all the various local languages spoken by the refugees.

Regular ‘return information updates’ were provided to all the refugees in all the settlements in the three districts. The information updates focussed mainly on security situation in the country of origin - South Sudan as this was considered an essential precondition for repatriation. In addition, refugees were informed about their legal status based on their decision to return or not. Those who choose to return were informed about the assistance they would receive prior to and during repatriation, and on arrival in South Sudan. As a key respondent stated;

*“We provided the refugees in all the settlements with regular information updates regarding security condition in Sudan. In addition, more information was provided regarding their status based on their decision whether to return or not. Refugees who were unwilling to return were informed of their rights and the choices available to them*.” (KII-UNHCR)

Further, information was provided about the general socio-economic conditions and the availability of social services in the areas of origin including health and education services. This information was supplemented by cross boarder visits made by members of Government of Sudan to the refugee communities in the settlements. These provided more information and assurance to the refugees.

*“While we tried to inform the refugees and provide information of conditions in south Sudan, we were limited to what was at our disposal. There were visits made by members of the government of Sudan that provided more information. Our roles and actions were limited to the information we received from our colleagues across the border”* (KII-UNHCR)

Secondly, UNHCR and the GoU and South Sudan facilitated confidence building visits. The visits termed: “Go visit and see” and “Come and inform” enabled refugees make self-assessments of the conditions in their country of origin. Representatives from the refugee community were selected from the various settlements in all three districts to participate in these visits. Representatives included settlement leaders, youth, and women. The delegations visited various locations and considered the security and socio-economic conditions. Upon return, the refugee representatives provided feedback to their peers who remained in the settlements. The visits enabled the refugees obtain detailed information about conditions in their country of origin as opposed to the general information disseminated.

*“We, together with all the other partners organised for refugees to go and visit their places of origin. We selected people from the settlements to represent various groups, women, leaders and others. With the support of the partners they went and visited their homes and came back and informed those who stayed in the settlements”* (KII-OPM)

### Steps undertaken during the repatriation process

First the UNHCR and the OPM determined and documented the official refugee status of all returnees. All the refugees who had decided to return provided written or signed a consent form. A “confirmation list” was generated and circulated and everyone had to append their name confirming their intention to return. The refugees who had provided consent of their willingness to be repatriated were issued a certificate of repatriation by the OPM. The UNHCR with the help of other partners generated a “pick up list”. Before departure, refugees handed over their refugee attestations and ration cards. Upon confirmation of those to be repatriated, arrangements were made for transference to the “repatriation centre”. Transportation arrangements were made for returnees by UNHCR. Returnees were transported in buses, while their luggage was loaded onto trucks. Each returnee was given a luggage allowance of up to 50 kilograms. Returnees were allowed to take with them domestic animals; however, livestock was not permitted because of logistical reasons.

Furthermore, the unit of repatriation was defined as an individual refugee. Registration for repatriation was therefore based on an individual's consent to return. However, during repatriation, the family was transported as a unit. Therefore, individuals who had consented to the repatriation exercise were registered under one household. Exceptions applied to all minors (under 18 years) who could only be repatriated in the company of an adult. As indicated by a key informant,

*“We decided to use an individual as the unit repatriation to ease planning. However, individuals were grouped as house holds to ensure they are able to move as a family. When repatriating we plan according to the number of individuals. There are members of the same household who chose to remain behind. Its voluntary repatriation and no one is forced to return.”* (KII-UNHCR)

Second, a repatriation centre was established at Dzaipi in Adjumani district. The centre acted as a transit point between the settlements where refugees lived and the reception centre in Nimule, South Sudan. Once a refugee(s) had decided to return, they would be transported from the settlements to the repatriation centre. At the centre, the refugees typically spent a day. Several services were provided at the repatriation centre including shelter, food (hot meals were served three times a day) and health care screening were conducted on every returnee prior to the journey to South Sudan.

Third, every individual on the “confirmation list” for return had to undergo mandatory medical screening at the health facility. Screening procedures carried out included general physical examination and diagnosis of acute and chronic health conditions including malaria and tuberculosis. Based on the screening results, travel restrictions were applied to individuals who were deemed medically unfit to make the journey back on the scheduled dates. Travel restrictions generally apply to the injured individuals, those with chronic illnesses, pregnant women of up to 7 months as well as mothers and their new-born babies. Those found sick were immediately put on treatment and advised not make the journey back. A new travel date was organised. During the journey, a “repatriation nurse” accompanied the returnees in an ambulance up to the reception point in Nimule, South Sudan.

Fourth, upon arrival in South Sudan, the returnees were received by a team consisting of UNHCR staff, government officials and other partners at the reception centre. Several services were provided at the reception centre including cross checking the identities of all returnees including names, age, home origins, resettlement assistance, and organising transportation to their homes. Further assessment was made of returnees' health status as they prepared to be resettled. Based on the returnee's destination, a repatriation convoy was organised. Returnees were often grouped according to their home origin to facilitate easier transportation. Many returnees, however, arranged to travel to their homes independently. In the event that any returnee did not know their homes or had no contacts, efforts were made by the UNHCR to trace their next of kin.

At the reception centre in Nimule, returnees received various forms of assistance from the UNHCR and other international organisations. Assistance included material and financial assistance were provided including food security items such as seeds and farm implements. Food assistance provided at the start of the return program extended for a period of between 3 – 6 months. Cash allowance was established, and each returnee received a cash grant of $50 per person (paid in south Sudanese pounds). Individuals who preferred to organise their own travel to places of origin were provided a travel grant based on the distance to their destination. At the return sites refugee reintegration process continued with support from various aid agencies. The UNHCR through various implementing partners continued to provide rehabilitation services to the returnees. The UNHCR and other partners in South Sudan provided returnees with livelihood support items including tailoring, oxen and ox ploughs, or rental of tractors plus seeds and tools.

Fifth, several stakeholders participated in the repatriation exercise based on the technical competences and the legal framework provided for in the tripartite agreement. The relief organisations included the UN agencies, NGOs, and District Local Governments. The UNHCR took the lead role in promoting and facilitating the repatriation exercise and supporting inter-agency cooperation with logistical and financial support. Additionally, the UNHCR provided food assistance, health, and medical supplies during the repatriation exercise. Other institutions included the governments of Sudan and Uganda, and local governments (districts) in the refugee hosting districts. Each organisation had defined roles and responsibilities during the repatriation process.

Refugee communities actively participated in the repatriation process. Refugee community representatives were selected from all the settlements in the three west Nile districts to promote and facilitate the repatriation process. These included refugee leaders, women and youth leaders. With assistance from the UNHCR and other partners, refugee community leaders conveyed messages of the need to go back home to fellow refugees in the settlements. The representatives collected the views, expectations, and concerns of refugees and relayed them to the organisations implementing the repatriation exercise. A respondent indicated.

*“We encouraged the active participation of all refugee communities during the repatriation exercise. The leaders of various groups settlements, young people and women participated in promoting the repatriation exercise in various ways including, conducting confidence building visits, and even deciding to go back home”* (KII-OPM)

The repatriation of Sudanese refugees took place during 2005 to 2009. A total of 94,578 refugees from 25,212 households were repatriated during the period as shown in Table 1. The highest number of refugees were repatriated during 2008 (41,927) 44.3% and (29,717) 31.4% during 2009, respectively.

**Table 1:** Trend of South Sudanese refugees repatriated from Uganda to South Sudan, 2005 – 2009

Year	Households	Individuals (%)
2005	85	212 (0.2)
2006	1,517	5,745 (6.1)
2007	6,580	17,189 (18.2)
2008	17,641	41,927 (44.3)
2009	7,565	29,717 (31.4)
**Total**	**25,212**	**94,578 (100.0)**

* Source: UNHCR planning and reports for Voluntary Assisted Repatriation of south Sudanese's from Uganda 2006 – 2009

Most of the refugees returned to the eastern Equatoria 55,808 (59%) and central Equatoria 38,711 (41%) districts as shown in Table 2. In central Equatoria, they returned mainly to Yei, Kajo-Keji, Juba, Lainya, Morobo districts, while in the eastern Equatoria, they mainly returned to Magwi, Torit, Nimule, Panyikwara, Owinykibul and Pajok districts.

**Table 2:** Destination and numbers of refugees repatriated

Province of origin	Destination (District)	Number of individuals N (%)
East Equatoria	Anikwara, Magwi, Nimule, Owinykibul, Pajok, Torit,	55,808 (59.0)
Central Equatoria	Juba, Kajo Keji Yei, Lainya, Morobo	38,711 (41.0)
**Total**		**94,519 (100.0)**

* Source: UNHCR planning and reports for Voluntary Assisted Repatriation of south Sudanese's from Uganda 2006 – 2009

**Figure 1:**
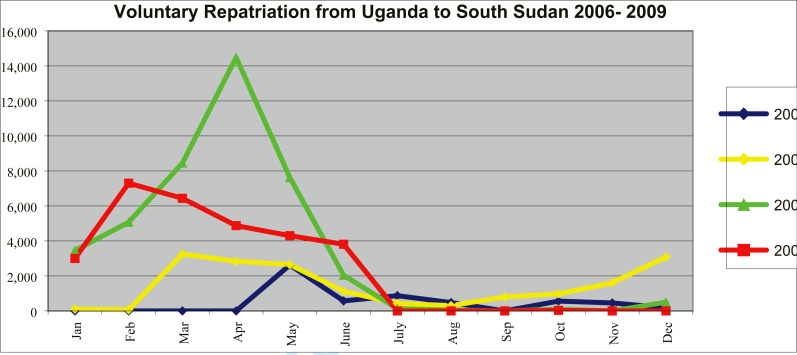
Trend of voluntary repatriation of refugees from Uganda to South Sudan, 2006 – 2009 * Source: UNHCR planning and reports for Voluntary Assisted Repatriation of south Sudanese's from Uganda 2006 – 2009

As shown in Figure 1, the repatriation of refugees from the west Nile districts Uganda mainly took place during the months of January to June each year. The peak period of the repatriation was April 2008 when an estimated 14,000 refugee were repatriated, while in 2009, an estimated 7,000 refugees were repatriated in the month of February.

### Non-Returnees

While most Sudanese refugees accepted and signed up to be repatriated, an estimated 10,000 opted to remain in Uganda. The majority 7,400 (82%) of those who stayed behind were based in Adjumani district, while others self-settled in several rural areas in the west Nile region. Those who stayed behind were mainly concerned about the security conditions and lack of social services in the areas of return. The majority preferred to return at such a time when conditions were considered safer. For the refugees who stayed behind, the OPM reassessed their status and continued to consider them as refugees for as long as they lived in government designated settlements. Security was provided for those who decided to remain in the settlements by OPM. Other provisions included access to a piece of land, social services including education and healthcare services. The UNHCR and the district local government provided assistance to those who chose to stay behind.

*“As a refugee agency, we considered all the concerns of all refugees. While the vast majority opted to return, others choose to stay in Uganda. Many of these had concerns over the security condition in areas of return. For those that did not want to return, arrangements were made including consideration of status of refugees, settlement and assistance*.” (KII-OPM)

Phase out strategies was developed by the UNHCR and the district local governments in the three districts. With the departure of most of the refugees, the UNHCR scaled down refugee assistance. Service delivery in the districts was guided by the Self-Reliance Strategy. The services for the refugees were integrated into the host population service delivery structures. This was meant to eliminate the existence of parallel service delivery structures. Refugee assistance assets including physical infrastructure mainly health facilities and water points e.g. boreholes were handed over to the district local governments.

## Discussion

The repatriation of south Sudanese refugees from Uganda was essentially voluntary. The process was facilitated through several interventions including involvement of several stakeholders including the UN agencies, government officials and the refugee communities, information campaigns about the repatriation exercise, facilitation and implementation confidence building visits “go and see and come and inform visits” by the refugees representatives, determination and documentation of the status of all returnees, and establishment of the repatriation centre. Furthermore, several services were provided to the returnees including health, socio-economic and legal advice prior to, during and in the aftermath of the repatriation exercise.

### Stakeholder's involvement in the refugee repatriation

Several stakeholders played essential roles during the process of repatriation. The different stakeholders included aid agencies, local and central government personnel as well as the refugee communities. These roles were stipulated in the framework of the “tripartite agreement” signed by the governments of Uganda and Sudan and the UNHCR. This ensured that return was ‘organised’ and not spontaneous. As observed by^20^, while the government of Mozambique promoted the return of refugees from Malawi to their home areas of origin, this was done against the approval of the UNHCR. In the end, inadequate participation and approval of the process restricted international aid, however this did not stop the repatriation process. Similarly, Tigrayan refugees were repatriated from Sudan in 1985 by the Royal Society of Tigray despite strong opposition from the UNHCR and the US Government^21^. These events disregarded internationally accepted policies for repatriation and presented various ethical and operational challenges. It is therefore essential to involve all key stakeholders and ensure clear roles and responsibilities at all operational levels in order to ensure an effective and efficient repatriation process.

### Involvement of the refugee community

The involvement of refugees is an important element of the repatriation exercise. In the case of the south Sudanese refugees hosted in west Nile district of Uganda, deliberate efforts were made to ensure that the refugees participated in the repatriation exercise. These included seeking and ensuring they provided consent to be repatriated, participated in confidence building visits to areas of origin and discussed the conditions of return. This practice was documented in other repatriation operations. As observed by^22^, while institutions play essential roles in facilitating the return process, the involvement of refugees is equally important in accepting the return programme. For instance^23^ observed that support provided to refugees to organise and directly participate in tripartite and other discussions regarding their future facilitated returns and enabled the process to be acceptable. In several repatriation programs, refugee involvement has been limited to only being informed that it was time to return. In East Timor and Cambodia, political influences led to repatriation strategies which accelerated refugee return in the absence of adequate preparation, and minimal consultation and participation^24^. According to^25^, inadequate participation by refugees in the repatriation process has had negative effects on the sustainability of returns.

### Information campaigns

The study findings emphasize the importance of sensitization and information campaigns in facilitating the repatriation process. In this study, information provided prior to and during repatriation enabled refugees decide freely whether or not to return. The motivation and decision to return is dependent on the information they receive about conditions in the country of origin. This is based on the evaluation of the accuracy of the information as well as evaluation of the conditions in the country of origin and status in the country of asylum^26^. Studies of the repatriation of Liberian refugees from Ghana^27^,^28^ and Mozambican refugees from Malawi^29^ showed the crucial role of information during the whole process. The promotion of voluntary repatriation through dissemination of re-assuring messages does encourage returns.

Information campaigns are a crucial prerequisite ‘to help ensure a free and informed choice regarding return’^30^. The information campaigns therefore ensured free and informed choice by refugees on the decision to return. These conditions are emphasised by the UNHCR noting that information campaigns ‘must be objective, accurate and neutral’ and it ‘is not propaganda, and care must be taken not to paint an overly rosy picture of the return’^31^. However, this study found that that information provided was incomplete and sporadic in some instances. Not all information was readily available. For example, information on security and socio-economic and political situation in places of origin was not disseminated as often. While we could not obtain views of returnees, it is essential that information provided to refugees is credible and comprehensive in order to demystify any fears, distrust and suspicions and encourage returns.

### Support provided to returnees

The support provided to the returnees prior to, during and after repatriation were essential towards facilitating their return and health and welfare. In this study we found that Sudanese refugees were provided with health services and other essential material assistance including livelihood implements and financial support. A study by^32^ highlighted the role of different forms of assistance that influence returns. The different services offered before, and during the repatriation exercise contributed towards a seamless reintegration process which is important for realising lasting solution to refugee problems. It is therefore essential to consider providing assistance, and other supportive services, to ensure effective resettlement of refugees. This is to enable returnees to become self-sufficient as early as possible upon return to their country of origin.

## Limitations

The study was conducted was several years after the actual repatriation. This meant that several key actors who were involved in the process of the refugee repatriation could not be interviewed. These included the returnees, government officials and UNHCR staff in return sites in South Sudan. This was mainly due to the time lag between repatriation and when the study was conducted as well as the unstable security conditions in South Sudan and the difficulty in tracing individuals who were repatriated. However, we interviewed key personnel in the various key organizations including the UN and local governments in the three refugee affected districts that were involved in the repatriation process in Uganda.

## Conclusions and Recommendations

The study has highlighted several key interventions that were critical towards effective repatriation exercises. These include community sensitization and regular information updates on socio-economic and security conditions in the country of origin. Accurate, timely and comprehensive information sharing aids decision making on whether to return. Additionally, it is important to provide support services prior to, during and in the post repatriation periods. The health services provided during the process of reparation as well as the package of support provided to returnees soon upon return were essential towards promoting the health and welfare of the returnees. The role and leadership of UNHCR and the involvement of several stakeholders including the refugees are crucial towards effective management and implementation of the process of repatriation.

From a policy perspective, it is essential to ensure repatriation processes follow international standards. This should be based on the premise that conditions are safe and secure in the country of origin. This is not only a pre-condition for return, but a critical element of refugee's decision-making regarding return to their country of origin. Therefore, information campaigns and confidence building visits to places of origins have positive effects on refugee's decision making on whether or not to return to their countries of origin. Equally essential is the involvement of key stakeholders including the aid agencies, local government authorities, government in country of origin, and the refugee communities.

This paper has focused on the process of repatriation of South Sudanese refugees from Uganda. From a methodological perspective there is need for further research on the process of repatriation. The questions could be framed along various themes including: How does refugee repatriation provide closure for refugees, repatriates and those that stay? How does politics both in the host and country of origin and by aid agencies influence the process of refugee repatriation? How can we protect repatriation and other durable solutions from politics? How do returnees adjust to socio-economic life in final areas of return following repatriation? How does repatriation as durable solution address contemporary protracted refugee crises in developing countries?
